# Blackstar and the limit of prognosis

**DOI:** 10.1017/S1478951526103290

**Published:** 2026-08-10

**Authors:** Amilton dos Santos Júnior

**Affiliations:** Department of Psychiatry, School of Medical Sciences, State University of Campinas (FCM-Unicamp), Campinas, Brazil

“Where are we now?”

– David Bowie, *The Next Day* (2013)

This essay is not about David Bowie’s illness. His songs will not be read as illustrations of symptoms. Not out of delicacy, but as a matter of method. What is at stake here is the album *Blackstar* (Bowie 2016), released a few days before his death, and a song from the previous album, *Where Are We Now?* (Bowie 2013), in which he revisits his trajectory, the places he passed through when he lived in Berlin, and raises the question of where we actually are.

Taken together, the 2 albums are about finitude, about knowing one will die and about dying. This interval is a territory much visited by physicians, but the work of art inhabits it by a path that clinical speech does not travel. The work knows about this interval, and it knows without promising anything.

Although no one can say for certain, several medical specialties can estimate a person’s survival time. Even when that estimate is true, it only measures the time that remains. And it may appear to organize that time, but it does not. Each experience of one’s own finitude is singular.

According to Austin (1962), to say is also to do: all speech is, to some degree, an act. A speech act can be true and still fail. Austin calls this infelicity, the failure that is not falsehood. A prognosis about the end of life is such a case: the estimate may be true and the act may still fail, because it promises to organize a time that it does not organize.

There are 2 durations that do not coincide: lived time and measured time. This distinction is at the center of the phenomenology of temporality. Measured time may be the same, yet the perception of its passing changes according to what is lived within it. The psychiatrist Minkowski (1970) made this reasoning a clinical concept, already thinking about the importance of lived time within psychopathology. An afternoon spent by an ailing grandfather with his grandchild will surely pass faster than an afternoon of laboratory and imaging tests. Prognostic expectation delivers measured time, but not lived time.

Psychiatrists do not weigh in on prognosis, but they stay alongside, often in silence, present in the face of terminality. In that space, they try to work where speech has already failed. And it is in that same interval that Bowie’s terminal work situates itself.

*Where Are We Now?* was released on 8 January 2013, on Bowie’s birthday, opening the album *The Next Day* after nearly 10 years without recording, when many had already considered him finished. It is not a song about death, but about revisiting. It lists places in Berlin where Bowie lived, Potsdamer Platz and the Dschungel, as a way for the self to locate itself in the geography of its own memory. From those places, he asks where we are now. It is the last moment in which that question can still be answered by looking back.

On that same 8 January, in 2016, he released his final album, *Blackstar*, which curiously does not bear that name in words but is a black star drawn on the cover. Two days after the release, on 10 January 2016, he died, and that 2-day interval is part of what makes *Blackstar* an act that is carried out. Where prognosis fails, the work accomplishes what it states. If *Where Are We Now?* asked while looking back, *Blackstar* answers from a new place, the end. In one of the songs, titled *Lazarus*, Bowie chose to name it after the figure of one who returns from the tomb, at the very moment he himself was dying. The same operation of locating oneself, previously done through the places of Berlin, is now done at the end. Bowie worked until his final days, and made his own death the matter of his last work. There is no way to describe death, but he stages it for the public in his final work.

The work is not the frail and weakened body, but the way the farewell unfolds. This does not mean it says better what medicine says poorly. It means it knows something else, of an order that the speech of health professionals does not reach through any technical or even humanistic refinement, however prepared one may be before these final moments. The work does not describe the farewell from outside: it shows it and it enacts it.

In a seemingly senseless lyric that joins the Nadsat of *A Clockwork Orange*, the language invented by Burgess, to Polari, the British gay argot, the album presents *Girl Loves Me*. It is an angry song, in which time comes undone, and language itself comes undone with it, deliberately near-incomprehensible. In *Dollar Days*, there is a phonetic pun between *I’m dying to* and *I’m trying to*, dying and trying spoken in the same breath, and there are echoes of *Where Are We Now?* in the English landscape the song gives back. The album closes with *I Can’t Give Everything Away*, the last track of the last record. It is the final act of locating oneself, now between what is given and what is kept silent.

The gift Bowie gave the public was a deliberate gesture, not a spectacle. He signed these acts and chose the exact moment to deliver them. The person Bowie keeps his privacy, but the artist hands over the close of his legacy. The work proves one thing only: that there is a way of knowing the interval between knowing and the end that does not pass through the speech that measures it.

That silence is being alongside, not leaving unattended. The insufficiency is not the professional’s, nor that of technique or sensitivity: it belongs to speech, which measures time and does not reach the way time is lived. For this reason the task is not to speak better, nor to find the exact word. Silence is the presence that remains.

Art does not teach us to die, just as it does not teach us to live, but it brings us closer to the unnameable. What it does is give form to the interval and show it inhabited. It marks the limit of what clinical speech can do, and marks it from within, inhabiting it, rather than describing it from outside. Art is the mode of inquiry that preserves what inquiry itself cannot exhaust. What remains is the question of the song, which is also that of every patient before the end: where are we now?
